# Soil seed burial and competition with surrounding plants determine the emergence and development of seedling of an endangered species *Horsfieldia hainanensis* Merr. in China

**DOI:** 10.1038/s41598-019-54644-7

**Published:** 2019-11-29

**Authors:** Xiongsheng Liu, Yinghui He, Yufei Xiao, Yong Wang, Yinghong Jiang, Yi Jiang

**Affiliations:** 1 Guangxi Key Laboratory of Superior Trees Resource Cultivation, Guangxi Zhuang Autonomous Region Forestry Research Institute, Nanning, 530002 P.R. China; 2 Department of Wildlife Protection, Department of Forestry of Guangxi Zhuang Autonomous Region, Nanning, 530022 P.R. China

**Keywords:** Ecology, Plant ecology

## Abstract

Three well-conserved *Horsfieldia hainanensis* Merr. populations were used to investigate their soil seed bank and seedling regeneration characteristics and their relationship to environmental factors. The results showed that the seed reserves were low in the *H*. *hainanensis* soil seed bank (16.93~24.74 seed/m^2^). The distribution pattern for the seeds and seedlings in the *H*. *hainanensis* populations was aggregated, and they were mainly found around 2–3 m from the mother plant. The seeds in the litter layer and the 5–10 cm soil layer showed no vigor, and only 25.7%~33.3% of the total seeds in the 0–5 cm soil layer were viable affected by the high temperature and humidity, the animals’ eating and poisoning. Affected by the height and coverage of the surrounding herbaceous layer and shrub layer, the seedlings of *H*. *hainanensis* could not obtain enough light and nutrients in the competition, resulting in the survival competitiveness of 1- to 3-year-old (1–3a) seedlings in the habitat had been in a weak position and a large number of seedlings died. It would take at least four years for seedlings to develop under the current environmental constraints. It can be concluded that the low seed reserve in the soil seed bank and high mortality of seedlings of *H*. *hainanensis* lead to slow or even stagnation of population regeneration, which was an important reason for the endangered of *H*. *hainanensis*. Therefore, the next research focus is to explore the influence mechanism of environmental factors on seed germination and seedling growth of *H*. *hainanensis*.

## Introduction

An important turning-point in the life cycle of a plant population is when the seeds in the seed bank germinate into seedlings and replenish the population. Two main factors affect the quality of this process: one is the characteristics and quality of the seeds in the soil seed bank, such as life span, size, maturity, and dormancy properties; and the other is the effect of population habitat conditions on the survival and growth of the seedlings^1^. The soil seed bank refers to the total number of active seeds in the soil litter layer and mineral soil. It is a key source of population renewal and plays an important role in population regeneration and succession, especially after disturbance events^2^. Seedling regeneration is the process of seedling formation through propagation, which is a crucial step in plant community succession^3^. The soil seed bank is the basis of seedling regeneration. Therefore, studying the soil seed bank characteristics and seedling regeneration will reveal the mechanism and trends behind population succession and can guide the management of vegetation. Such studies are important during the restoration and expansion of endangered plant populations^4^.

*Horsfieldia hainanensis* Merr. belongs to the Myristacaceae, and is a unique tall evergreen tree that grows in a restricted area of China. The plant is mainly distributed in Guangxi, Yunnan, and Hainan provinces, and is found mostly in shady and wet valleys at an altitude of 400–450 m along the border between China and Myanmar/Vietnam. It is a landmark species for wet tropical rainforest. Therefore, it is important to study the floristic composition, geographical distribution, ecological habits, and conservation biology of these endangered plants in tropical rainforest areas^5^. The *H*. *hainanensis* habitat has recently become significantly fragmented due to its own biological characteristics, as well as to human destruction, and illegal logging, which have all led to slow and declining population regeneration. Therefore, it has been listed as a grade 2 key protected plant and endangered tree species in China^6^. Population decline and slow regeneration are generally caused by severe seed depletion or obstacles to seedling regeneration in the population^7^. The field investigation showed that seedlings under the natural forest were few and poorly growing, and young trees were extremely rare in the natural forest of *H*. *hainanensis*. We speculate that the reason why it is difficult for seedlings of *H*. *hainanensis* to develop into young trees is that the influence of environmental factors such as soil, light and surrounding vegetation, which result in the slow regeneration of the population. Therefore, in order to investigate the decline and slow regeneration of the *H*. *hainanensis* population, we explored the soil seed bank, seedling regeneration, and their relationship to the *H*. *hainanensis* natural environment so that any reasons underlying the population decline, such as a small soil seed bank and low seedling regeneration, could be identified. The results provide a scientific basis for the protection, restoration, and expansion of the *H*. *hainanensis* population.

## Results

### *H*. *hainanensis* seed reserves and spatial distribution in the soil seed bank

The number of seeds and the proportion of seed viability in seed bank of the three *H*. *hainanensis* populations in the different soil layers are shown in Table 1. The differences between the seed reserves in the soil seed bank among the three populations was significant (*P* < 0.05) with the largest (24.74 seeds/m^2^) and smallest (16.93 seeds/m^2^) seed reserves being observed in population of Niandou Village, Daxin County (PND) and population of Sanlian Village, Longzhou County, Guangxi (PSL), respectively. The layer containing most of the seeds was the litter layer in all three populations, and accounted for 70.81%~75.68% of the total seed reserves. This was followed by the 0–5 cm soil layer. However, the 5–10 cm soil layer contained hardly any seeds. The seed vigor analysis indicated that all seeds found in the litter and 5–10 cm layers had lost their vigor. Only 25.7%~33.3% of the seeds in the 0–5 cm layer were still viable.

**Table 1:** Number of seeds and the proportion of viable seeds in the *H*. *hainanensis* seed bank in the different layers.

Population	Total seed reserves (seeds/m^2^)	Seeds in the different layers (%)		
		Litter layer	0–5 cm soil layer	5–10 cm soil layer
PSL	16.93 ± 1.58a	75.68 ± 1.85a (0)	24.32 ± 1.85a (25.7)	0.00 ± 0.00a (0)
PNG	18.75 ± 1.63a	70.81 ± 0.62a (0)	25.10 ± 1.97a (27.3)	4.09 ± 2.52ab (0)
PND	24.74 ± 0.69b	74.83 ± 2.60a (0)	17.83 ± 3.19a (33.3)	7.34 ± 0.89b (0)
F value	8.904	1.926	0.745	5.668
*P*	0.016	0.226	0.514	0.041

Note. The values represent mean ± SE. One-way ANOVA with the Duncan post hoc test was used to determine significant differences. Within a column, mean values with different letters are significantly different (*p* < 0.05). Numbers in parentheses indicate the percentage of viable seeds. PSL: population of Sanlian Village, Longzhou County, Guangxi; PNG: population of Nonggang Nature Reserve; PND: population of Niandou Village, Daxin County.

**Figure 1:**
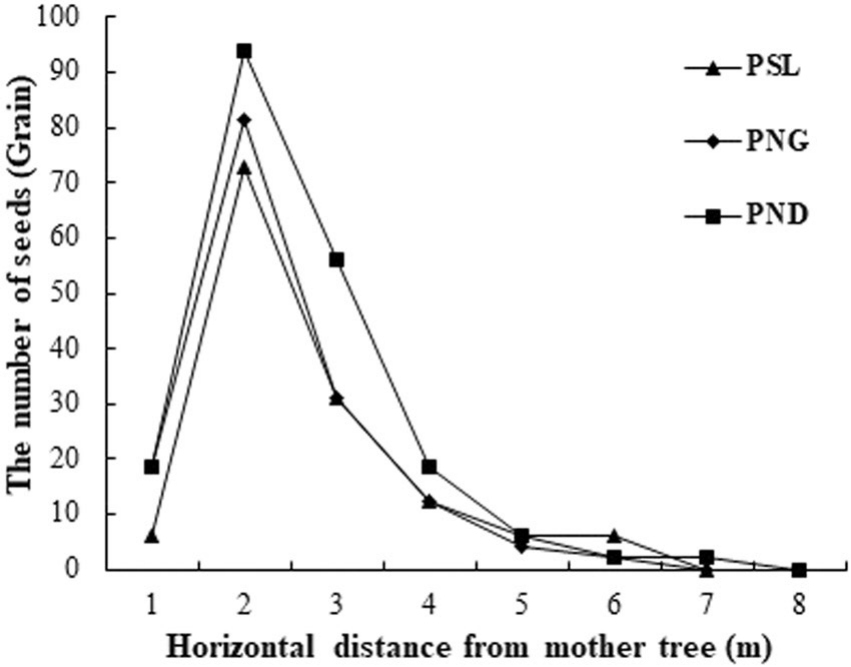
Seed numbers and horizontal distribution in the *H*. *hainanensis* seed banks. Note. PSL: population of Sanlian Village, Longzhou County, Guangxi; PNG: population of Nonggang Nature Reserve; PND: population of Niandou Village, Daxin County.

The horizontal distributions of the three *H*. *hainanensis* soil seed banks are shown in Fig. 1. Most of the seeds were located 2–3 m from the mother plant, but the seed number decreased gradually as the distance increased from the mother plant, which meant that there were almost no seeds in the soil under the edge of the crown.

### Seed distribution pattern in the *H*. *hainanensis* soil seed bank

The seed distribution patterns of the three *H*. *hainanensis* populations are shown in Table 2. The diffusion coefficient (*C*) and agglomeration index (*PAI*) were greater than 1, the aggregation degree (*I*) and *C*_α_ indicator were greater than 0, the mean crowding was larger than $\bar{X}$, and the K indicator of the negative binomial distribution was greater than 0, but less than 8 in all three populations, which indicates that the seeds had a clustered distribution in all three populations.

**Table 2:** Distribution pattern of the *H*. *hainanensis* soil seed banks.

Population	$\bar{X}$	*C*	*I*	M^*^	*PAI*	*C*_α_	*K*	Result
PSL	0.6771	1.2627	0.2627	0.9397	1.3879	0.3879	2.5778	Clustered
PNG	0.7500	1.5494	0.5494	1.2994	1.7325	0.7325	1.3652	Clustered
PND	0.9896	1.5780	0.5780	1.5675	1.5840	0.5840	1.7122	Clustered

Note. PSL: population of Sanlian Village, Longzhou County, Guangxi; PNG: population of Nonggang Nature Reserve; PND: population of Niandou Village, Daxin County. $\bar{X}$: average population abundance, *C*: diffusion coefficient, *I*: aggregation degree, M^*^: mean crowding, *PAI*: agglomeration index_,_ *C*_α:_ *C*_α_ indicator, *K*: negative binomial distribution.

### Seedling regeneration and its spatial distribution characteristics

**Figure 2:**
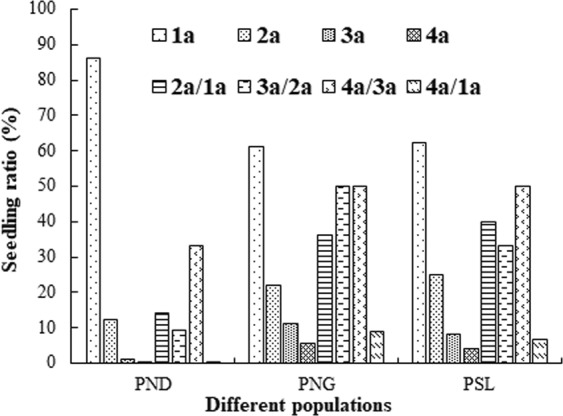
*H*. *hainanensis* seedling ratio and seedling transformation in the different populations. Note. 1a: 1-year-old seedling, 2a: 2-year-old seedling, 3a: 3-year-old seedling, 4a: 4-year-old seedling, 2a/1a: the transformation rate from 1-year-old to 2-year-old seedling, 3a/2a: the transformation rate from 2-year-old to 3-year-old seedling, 4a/3a: the transformation rate from 3-year-old to 4-year-old seedling, 4a/1a: the transformation rate from 1-year-old to 4-year-old seedling.

The *H*. *hainanensis* seedlings usually appear in June, with emergence reaching its peak in July, and ceasing in August. The seedling proportions and transformation rates for *H*. *hainanensis* in the three populations are shown in Fig. 2 and Supplementary Dataset 1. The highest percentage was 1-year-old seedlings, which accounted for 86.3%, 61.1%, and 62.5% of the seedlings in PND (population of Niandou Village, Daxin County), PNG (population of Nonggang Nature Reserve), and PSL (population of Sanlian Village, Longzhou County, Guangxi), respectively. The lowest percentage was 4-year-old seedlings, which accounted for 0.4%, 5.6%, and 4.2% of the seedlings in PND, PNG, and PSL, respectively. The transformation rate from 1-year-old to 4-year-old seedlings (4a/1a) was relatively low at 0.4%, 9.1%, and 6.7% in PND, PNG, and PSL, respectively. Therefore, the probability of seedling survival and their development into saplings was minimal.

**Figure 3:**
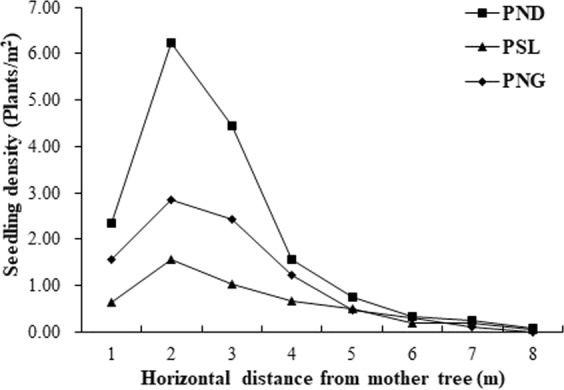
Distribution of *H*. *hainanensis* seedlings in the different populations. Note. PSL: population of Sanlian Village, Longzhou County, Guangxi; PNG: population of Nonggang Nature Reserve; PND: population of Niandou Village, Daxin County.

The seedling distribution of the three *H*. *hainanensis* populations is shown in Fig. 3. The distribution of the seedlings was consistent with the soil seed bank. The results showed that the distribution was concentrated within 2–3 m of the mother plant and that the number decreased as the distance from the mother plant increased. The seedling number was close to 0 at the edge of the crown. There are significant variations in the seedling numbers at the same relative location among the three populations. The PND seedling numbers were greater than the PNG and PSL numbers in the corresponding regions, and the PNG seedling numbers in the first four plots were all greater than PSL, but were slightly less than PSL in the last four plots.

### Relationship between the spatial distribution of the seedlings and microhabitat

**Figure 4:**
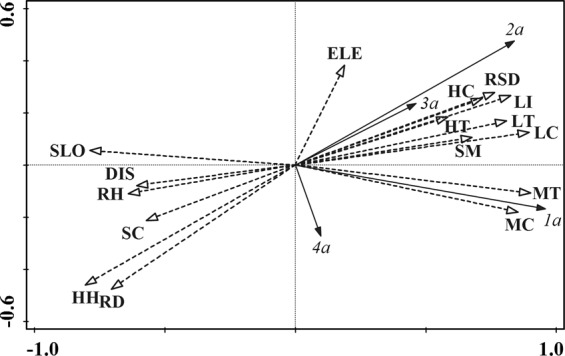
Redundancy analysis (RDA) ordination diagram of *H*. *hainanensis* seedlings and the microhabitat variables. Note. MC: moss layer coverage, MT: moss layer thickness, LC: litter coverage, LT: litter thickness, SM: soil moisture, HT: humus thickness, LI: light intensity, HC: herb coverage, RSD: root disc layer depth, ELE: elevation, SLO: slope, DIS: the distance from the plot center to large trees that were within 5 m of the center., RH: air humidity, SC: shrub layer coverage, HH: herb height, RD: rock bareness.

The RDA ordination of the seedling distribution and microhabitats (Fig. 4) indicated that the distributions of one-year (1a), two-year (2a) and three-year (3a) -old seedlings were positively correlated with the moss layer coverage (MC) and thickness (MT), litter coverage (LC) and thickness (LT), soil moisture (SM), humus thickness (HT), light intensity (LI), herb coverage (HC), root disc depth (RSD), and elevation (ELE), but negatively correlated with slope (SLO), the distance to surrounding big trees (DIS), air humidity (RH), shrub cover (SC), herb height (HH), and rock bareness (RD). The distribution of 4-year-old seedlings (4a) had a poor relationship with microhabitat variables, which suggested that the effect of microhabitat on 4a seedlings was less significant, probably because these seedlings have survived any initial environmental challenges.

The principal component analysis (Table 3) showed that the environmental factors were highly correlated with the growth of 1–4a seedlings. Of these, MT (0.886), LC (0.880), MC (0.840), and LI (0.810) were the four major positive factors, whereas HH (−0.789), SLO (−0.773), RD (−0.690), and RH (−0.628) were the four main negative factors.

**Table 3:** Correlation coefficient matrix for seedling abundance and environmental factors.

Environmental factor	Species ordination axis		Environment ordination axis	
	SPEC AX1	SPEC AX2	ENVI AX1	ENVI AX2
ELE	0.184	0.342	0.188	0.385
SLO	−0.773	0.014	−0.787	0.012
HC	0.703	0.263	0.716	0.296
HH	−0.789	−0.440	−0.803	−0.495
SC	−0.560	−0.217	−0.570	−0.244
MC	0.840	0.131	0.855	−0.147
MT	0.886	0.059	0.902	−0.067
LC	0.880	0.152	0.895	0.171
LT	0.795	0.180	0.809	0.203
HT	0.573	0.201	0.583	0.226
RD	−0.690	−0.451	−0.703	−0.508
RSD	0.750	0.288	0.763	0.324
LI	0.810	0.278	0.824	0.312
RH	−0.628	−0.132	−0.639	−0.148
SM	0.665	0.115	0.677	0.130
DIS	−0.596	−0.078	−0.607	−0.088

Note. ELE: elevation, SLO: slope, HC: herb coverage, HH: herb height, SC: shrub layer coverage, MC: moss layer coverage, MT: moss layer thickness, LC: litter coverage, LT: litter thickness, HT: humus thickness, RD: rock bareness, RSD: root disc layer depth, LI: light intensity, RH: air humidity, SM: soil moisture, DIS: the distance from the plot center to large trees that were within 5 m of the center.

## Discussion

The seed reserves in the soil seed banks of the three *H*. *hainanensis* populations were low (16.93–24.74 seeds/m^2^), and the seeds are mainly concentrated in the litter layer, accounting for 70.81%–75.68% of the total seed reserves. Only a small amount of seeds is distributed in the 0–5 cm and 5–10 cm soil layers, and only 25.7%–33.3% of the seeds in the 0–5 cm soil layer were vigorous. Studies on the soil seed banks of *Artocarpus hypargyreus*^8^ and *Amygdalus mongolica*^9^ indicated that the seed reserves in the soil seed bank of both species were low (23.3~31.9 seeds/m^2^ and 2.6~21.2 seeds/m^2^, respectively) due to animal feeding, and human and environmental factors. This had led to slow population regeneration and they have also been given endangered status, which is consistent with our results. The seed setting rate for *H*. *hainanensis* is low (only 6.64%)^10^. The fruit of *H*. *hainanensis* is large (the weight of a single fruit is 47.63–75.39 g, the longitudinal diameter of the fruit is 71.09–85.20 mm, and the transverse diameter of the fruit is 40.90–45.20 mm), and it mainly depends on the gravity to propagate^10^, as a result, it is difficult to pass through the isolation zone formed by the coiled layer of the root system or litter layer under the forest. Therefore, the seeds in the soil seed bank are mainly concentrated in the litter layer. While the seeds of *H*. *hainanensis* transported by animals and rainwater result in a small amount of seeds distributed in 0–5 cm and 5–10 cm soil layers^11^. According to our field observation, the seeds of *H*. *hainanensis* mature in summer, and the time interval from the beginning of seed diffusion to its complete loss of vitality is less than 3 months. The seed bank has a short duration, which belongs to the instantaneous seed bank^12^, and cannot be stored for a long time under natural conditions. This may be because *H*. *hainanensis* is distributed in the shade and wet valley, the region suitable for seed germination is limited, and most of the seeds are distributed in the crown width of the mother tree, which shows a clustered distribution. It increases the competition among seeds, and that affected by the storage environment, such as high temperature, high humidity and so on. The seed cells have strong respiration and produce a lot of H_2_O, positive feedback regulating the cell respiration, and which consumes a lot of stored nutrients in the seed^13^. In addition, The seeds of *H*. *hainanensis* have a thick seed coat (4 mm)^10^, and are easy to rot under high temperature and high humidity, which will produce poison to the seeds. The seed aggregate distribution will aggravate the spread of the poison, which result in the loss of the seed vigor and low germination rate. At the same time, the gnawing of insects, ants, birds and animals, the impact of surface runoff, the wind and other factors have greatly reduced the intact seeds in the soil seed bank^14^. The reason why 25.7–33.3% seeds are active in the 0–5 cm soil layer are may be that the seeds in the 0–5 cm soil layer effectively avoid the impact of insects, ants, birds, animals and other factors such as gnawing, surface runoff and wind. Moreover, the seeds buried by soil, have less contact with the air, the internal respiration of the seeds is weak consequently, which can maintain the internal metabolic balance to a certain extent^15^. The specific reasons need to be further studied.

Seedlings are a key part of the population and an important stage in the plant life cycle. The plant population can only be successfully dispersed and maintained through seedling regeneration^16^. Our study on seedling regeneration and the spatial distribution of three *H*. *hainanensis* populations indicated that a majority of the seedlings lived within 2–3 m of the mother plant, and that mortality rates were high during seedling growth, which resulted in limited numbers of older seedlings (the proportion of 4-year-old seedlings is only 0.4%~5.6%) and a low transformation rate (the transformation rate from 1-year-old to 4-year-old seedlings (4a/1a) was 0.4%~9.1%). Swamy^17^ showed that host-specific natural enemies, such as specific herbivores and pathogens, were easily transmitted from adult plant individuals to nearby offspring, and that the seeds of many different species are usually dispersed around the mother plant, which leads to a high seedling density. However, this facilitates the propagation of specific herbivores and pathogens, which leads to a high seedling mortality rate. Therefore, Swamy believed that the seedling pattern of the population was generally skewed or was located far from the mother tree. However, other researchers have demonstrated that the regeneration of seedlings close to the mother tree is inhibited due to the constraints of the mother tree. Natural plant regeneration has a population supplement curve that varies with the distance from the mother tree, and the plant regeneration survival rate is elevated until stabilized by the increase in distance. This stabilizing distance is defined as the critical escape distance for plant population regeneration, and the seed or seedling regeneration process within the escape distance is limited by the microhabitat factors under the mother tree^18–20^. Our results confirm this hypothesis. Studies have shown that the spatial heterogeneity of the understory habitats of a population plays an important role in the growth and distribution patterns of the seedlings^21–23^. Topography and elevation are the main sources of habitat heterogeneity and can lead to spatial changes in ecological factors, such as light, temperature, water, and soil nutrients. Variations in these ecological factors directly affect the spatial settlement and survival of the seedlings^24,25^. *H*. *hainanensis* grows in shaded wet valleys. Therefore, soil depth and illumination are limited. In addition, its fruits are large, which is not favorable for fruit landing where there is a significant slope. However, the seedling growth range is limited on a flat terrain. Air humidity, shrub, and herb restrictions mean that *H*. *hainanensis* seedlings are often not able to obtain sufficient light and nutrients. Therefore, the large *H*. *hainanensis* seedling mortality rate may be a joint consequence of mother tree distribution and microhabitat.

In summary, the limited seed reserves and seed vigor in the *H*. *hainanensis* soil seed bank and its highly clustered seed distribution pattern not only restricts seed germination, but also confines seedling growth. In addition, the survival competitiveness of the 1–3a seedlings was poor, and was significantly affected by the herb and shrub coverage. The seedlings needed at least four years to meet any environmental microhabitat challenges, which meant that longer term survival was limited and few seedlings developed into saplings. Therefore, the low transformation rate from seed to seedling and the high seedling mortality rate of the *H*. *hainanensis* population due to the limited seed reserves and vigor, the highly aggregated seed distribution, and the understory microhabitat, means that population regeneration is slow or even stagnant. This may be one of the most important reasons affecting the survivability of *H*. *hainanensis* populations.

## Materials and Methods

### General information of the experimental site

A preliminary investigation showed that there were three locations with large numbers of *H*. *hainanensis* seeds and seedlings. Therefore, these sites were selected as the experimental sites. The sites were Sanlian Village, Longzhou County, Guangxi (PSL), Nonggang Nature Reserve (PNG), and Niandou Village, Daxin County (PND). General information about these sites is shown in Table 4.

**Table 4:** Study site habitat conditions.

Sites	Longitude and latitude	Altitude (m)	Landforms	Soil	Distribution	Number of trees	Degree of artificial destruction
PSL	106.7958E 22.5772N	540	Peak cluster	Calcareous soil	Clustered islands	18	Mild
PNG	106.9426E 22.4848N	163	Peak cluster	Calcareous soil	Clustered islands	57	Severe
PND	106.7987E 22.7512N	375	Peak cluster	Calcareous soil	Clustered islands	56	Medium

Note. PSL: population of Sanlian Village, Longzhou County, Guangxi; PNG: population of Nonggang Nature Reserve; PND: population of Niandou Village, Daxin County.

### Soil seed bank and seedling distributions

**Figure 5:**
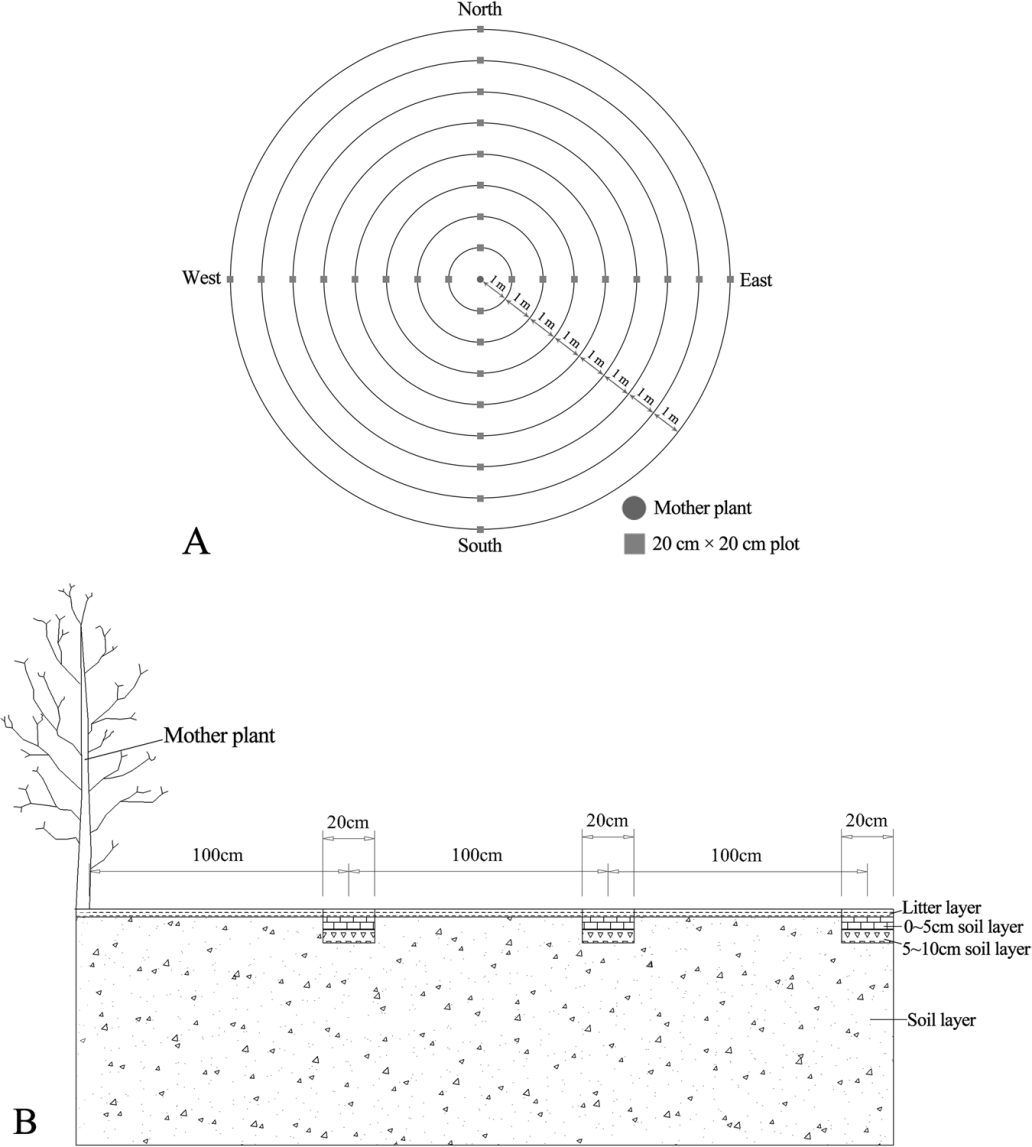
(**A**) Schematic diagram of plots. (**B**) Schematic diagram of soil plots collection.

The crown of a typical *H*. *hainanensis* tree is about 8.0 m × 8.0 m and its seeds are relatively large. The seeds are dispersed by gravity, which means that most of its seeds are scattered in the crown area. According to Bigwood^26^, in May 2016 (after the end of seed rain), three adult *H*. *hainanensis* plants with desired growth potential were selected from PSL, PNG, and PND, respectively. In the present study, The mother plant was at the center of 20 cm × 20 cm plots that were located east, west, south, and north of the mother plant. These plots were 1 m apart. A total of eight subplots were established in each direction, and soil samples from the litter layer, the 0–5 cm layer, and the 5–10 cm layer in each subplot were collected and screened, using a 0.25-cm sieve, to determine the seed number in the soil layers from each subplot (Fig. 5A,B). The 2,3,5-Triphenyltetrazolium chloride (TTC) staining method was used to determine seed vigor^27^, with the viable seeds representing the seed reserves and the distribution of the soil seed bank. In August 2016, with the mother plant at the center, sample circles with radii of 1 m, 2 m, 3 m,… 8 m were set outwards from the center, and the number of seedlings were determined in each sample circle (Fig. 5A). The seedling density was represented by the ratio of the total number of seedlings to the area of the corresponding sample circle (plants/m^2^).

The seed reserves in the *H*. *hainanensis* population were calculated using the sample plot seed data, and the vertical and horizontal distributions of the *H*. *hainanensis* seeds in the soil seed bank were analyzed. The aggregation index method was used to determine the distribution pattern for *H*. *hainanensis* seeds in the population, and the specific indicators were as follows:

1. Diffusion coefficient (*C*): ${\rm{C}}=\frac{{S}^{2}}{\bar{X}}$, where $\bar{X}$ indicates the average population abundance and *S*^2^ is the population abundance variance;
2. Aggregation degree (*I*): $$I=\frac{{S}^{2}}{\bar{X}}{\textstyle \text{-}}1$$;
3. Mean crowding (M^*^): $${M}^{\ast }=\bar{X}+\frac{{S}^{2}}{\bar{X}}{\textstyle \text{-}}1$$;
4. Agglomeration index (*PAI)*: $PAI=\frac{{M}^{\ast }}{\bar{X}}$;
5. *C*_α_ indicator: $${C}_{\alpha }=\frac{I}{\bar{X}}$$;
6. *K* indicator of negative binomial distribution: $K=\frac{{\bar{X}}^{2}}{({S}^{2}-\bar{X})}$;

A *C* and *PAI* of less than 1 indicates an even distribution, but equal to or greater than 1 represent random or aggregated distributions, respectively. When *I* and *C*_α_ indicators are less than 0, then the distribution is even, but when they are equal to or greater than 0, then the distribution is random or aggregated, respectively. When M^*^ is less than $\bar{X}$, equal to $\bar{X},$ or larger than $\bar{X}$, then the corresponding distribution is even, random, or aggregated, respectively. When K is less than 0, greater than 0, or greater than 8 then the distributions are even, aggregated, or random, respectively^28,29^.

### Microhabitat survey of seedling distribution

Following Nicotra^30^ and Molofsky^31^, In 2014, eight 5 m × 5 m temporary plots were installed in the PND, PNG, and PSL populations, The 1-year-old seedlings in the plots were tracked and observed for 4 consecutive years, and the mortality and preservation rate of seedlings were counted each year. The investigated environmental factors included three topographic factors (elevation, slope and rock bareness), nine vegetation factors (herb coverage, herb height, shrub layer coverage, moss layer coverage, moss layer thickness, litter coverage, litter thickness, humus thickness, and root disc layer depth), two meteorological factors (light intensity and air humidity), soil moisture, and the distance from the plot center to large trees that were within 5 m of the center.

Gradient lengths were obtained by trend correspondence analysis of the species-sample data using Canoco 5.0 software. The gradient length at the first axis was 2.649, which, being less than 3, meant that the linear ranking model could be used. A Monte Carlo test was applied to determine whether there were significant statistical correlations between the explanatory variables and the response variables. This information was used to clarify the variation interpretation of the microhabitat variables to response variables. The ordination was established using redundancy analysis (RDA)^32^.

## Supplementary information

Supplementary Dataset 1
